# News and Notes

**Published:** 1908-07

**Authors:** 


### NEWS AND NOTES

Dr. Jno. F. Denton made a combined professional and pleasure visit to Dalton, Ga., recently.

The death arte in the city of New York, for the week ending May 30, was the lowest for any week since 1895.

Dr. F. G. Hodgson paid a short visit to his old home in Athens during the recent commencement of the State University.

Dr. Henry Slack, of LaGrange, was in Atlanta for a short stay recently.

Dr. Edgar G. Ballenger and family are located at Lithia Springs for the summer months.

Dr. W. B. Armstrong was one of the fortunate ones who enjoyed the A. B. & A. excursion to Brunswick and the sea shore.

Dr. J. R. Brock, of Dade county, is in the city for several weeks, to attend the general asesmbly. Dr. Brock represents his county in the upper house.

The friends of Dr. W. W. Pilcher, of Warren county, are urging him to make the race for congress from his district.

Dr. Fannin has been appointed to the superintendency of Wesley Memorial Hospital—the place made vacant by resignation of Dr. Glenn.

A new quarterly medical journal has been established by Dr. Hendrick Stern, of New York, with the title ,'The Achives of Diagnosis." The second number has recently appeared and contains twelve original articles of distinct merit, and a general review of the current literature bearing upon diagnosis. The importance of this portion of medicine is so great that we feel safe in predicting success to Dr. Stern in his undertaking.

Dr. Glenn leaves in a few days to take up practice in Columbus, Ga. His many friends regret exceedingly to see him leave this section.

---

It will be a source of much gratification to the many friends of Dr. Roy Harris, to know that he is out again after his recent severe spell of typhoid.

---

Dr. R. T. Dorsey leaves soon for his vacation and to secure much needed rest after the hard work of the past winter and spring.

---

Dr. Floyd W. McRae has resigned from the staff of Grady Hospital. His successor has not yet been selected, but Dr. W. B. Armstrong is being prominently mentioned for the place.

---

In the death of Dr. Geo. Grimes, of Columbus, Ga., that community has suffered a very severe loss as he has been one of their foremost practitioners for some thirty years, as well as a leading citizen.

---

It is with deep regret that we read an account of the death of Dr. Benj. W. Bizzell, at Phoenix, Arizona, June 26. Dr. Bizzell was formerly a resident of Atlanta and for several years was a member of the board of health.

---

Dr. R. R. Kime, who has just returned from the Chicago, Rochester, (Ill.), and the University of Michigan Medical Association, and talks very interestingly of his visit to the Dr. Mayo.

---

Dr. E. C. Davis and Dr. E. G. Jones drove through the country to Athens, Ga., in the former's new four cylinder runabout. The entire trip was without mishap and both gentlemen report an enjoyable time.

---

Dr. E. Bates Block, whose marriage to Miss Julia Porter, of Atlanta, was one of the recent society events of that city, is still in Europe where he and Mrs. Block are enjoying quite an extended trip to the different places of interest.

We are glad to be able to report that Dr. Theo. Toepel is convalescing from a very severe spell of typhoid fever.

---

Dr. Joseph Mines an dfamily are visiting his father in Birmingham, Ala. Dr. Hines drove through the country in his automobile.

---

Since the resignation of the entire house staff of Grady Hospital, Dr. L. D. Pattillo has been serving as house surgeon. The duties of this position are by no means new to Dr. Pattillo, as he already holds certificate for somplete service from Grady Hospital.

---

A meeting of the Surgeons of the Atlanta, Birmingham and as he already hold certificate for complete service from Grady included the annual report of the officers, addresses by the physicians and surgeons employed by the road, and the election of officers. Dr. W. S. Elkin is chief surgeon of the road.

---

An effort is being made by each of the Atlanta Medical Colleges, to become the medical branch of the University of Georgia, and equally as strenuous effort being put forth by the Medical College of Augusta, to retain its position in this capacity.

---

Prof. Robert Koch is making an extended tour of the United States, where he is being warmly welcomed by the medical profession. His discovery of the tubercular bacillus has resulted in saving an enormous number of lives—probably more than all other medical discoveries combined. In the city of New York, alone, in 1881, numbered 6,123, while in 1906 there were only about 8,000 deaths in spite of the enormous increase in population.

---

At the commencement exercises of Tulane University, New Orleans, May 20, it was announced that the suit of the Newcomb heirs against Newcomb College had been decided in favor of the college, and that after six years of litigation, the supreme court of New York State, had declared that the $2,000,000 willed by Mrs. Newcomb would now become the property of Tulane for its Department of Women as soon as the formalities could be accomplished.

### THE RESEARCH DEFENCE SOCIETY.

A society has been formed in London called the Research Defence Society, whose object is to counteract the propaganda of the antivivisectionists who have been very active in England for the last few years. The society will give information to all inquirers, issue articles and pamphlets, send out speakers and lecturers, and carefully examine the truth of the teachings of the antivivisectionists with their misleading and calumnious statements. It is thought that the passive attitude of the medical profession in the past was a mistake and that it is time to wage an active campaign to show that vivisection has a prominent bearing on public health and welfare, to show the real nature of experimental vivisection, the benefits which it has conferred both to man and the lower animals, and the still greater advances that will be made possible by it in the future. The society will also demonstrate that the medical men who employ vivisection are not less humane than the rest of their countrymen. The society already has more than 800 members among whom are most of the leading physicians and scientists of London and also many well known persons in public life.

### ARMY MEDICAL DEPARTMENT EXAMINATIONS, 1908.

The Act of April 23, 1908, reorganizing the Medical Corps of the Army, gives an increase in that Corps of six colonels, twelve lieutenant-colonels, forty-five majors, and sixty captains or first lieutenants, and establishes a Medical Reserve Corps as an adjunct to the Medical Corps. Under this recent act, the lieutenants of the Medical Corps are promoted to captain after three years' service instead of five, and the increase in the higher grades insures promotion at a reasonable rate all through an officer's military career. Furthermore, applicants who are found qualified in the preliminary examination are appointed first lieutenants of the Medical Reserve Corps and ordered to the Army Medical School in Washington, D. C., for eight months' instruction.

### THE MEDICAL CORPS.

Preliminary examination for appointment in the Medical Corps will be held on August 3, 1908, and formal applications should be in possession of the War Department prior to July 1st. The applicant must be a citizen of the United States, between twenty-two and thirty years of age, a graduate of a medical school legally authorized to confer the degree of doctor of medicine, of good moral character and habits, and must have had at least one year's hospital training or its equivalent in practice. The examination will be held concurrently throughout the country at points where boards can conveniently be assembled, and due consideration will be given to the localities from which applications are received, in order to lessen the traveling expenses of applicants as much as posisble.

The examination in subjects of general preliminary education may be omitted in the case of applicants holding diplomas from reputable literary or scientific colleges, normal schools or high schools, or graduates of medical schools which require an entrance examination satisfactory to the faculty of the Army Medical School.

The large number of vacancies created in the Medical Corps by recent legislation makes it certain that all successful candidates will be recommended for a commission for several years to come.

### THE MEDICAL RESERVE CORPS.

It is desired to obtain and maintain a list of qualified medical men all over the country who are willing to serve as medical officers in time of emergency, and to such men the President is authorized to issue commissions as First Lieutenants, Medical Reserve Corps. It is recognized that it will be necessary to place only a limited number of these officers on the active list in time of peace, and it is hoped that young medical men throughout the country and medical officers of the militia of the various states may be sufficiently interested to secure positions on the Medical Reserve Corps list.

An applicant must be between twenty-two and forty-five years of age, a citizen of the United States, a graduate of a reputable medical school legally authorized to confer the degree of doctor of medicine, and must have qualified to practice medicine in the State in which he resides. Examinations will be held in the near future and will embrace the practical medical subjects.

Full information concerning the Medical Corps and the Medical Reserve Corps may be procured upon application to the Surgeon General, U. S. Army, Washington, D. C.

